# Antifungal Biofilm Inhibitory Effects of Combinations of Diclofenac and Essential Oils

**DOI:** 10.3390/antibiotics12121673

**Published:** 2023-11-28

**Authors:** Alexia Barbarossa, Antonio Rosato, Antonio Carrieri, Roberta Tardugno, Filomena Corbo, Maria Lisa Clodoveo, Giuseppe Fracchiolla, Alessia Carocci

**Affiliations:** 1 Department of Pharmacy—Pharmaceutical Sciences, University of Bari “Aldo Moro”, 70125 Bari, Italy; alexia.barbarossa@uniba.it (A.B.); antonio.rosato@uniba.it (A.R.); antonio.carrieri@uniba.it (A.C.); roberta.tardugno@uniba.it (R.T.); filomena.corbo@uniba.it (F.C.); alessia.carocci@uniba.it (A.C.); 2 Interdisciplinary Department of Medicine, School of Medicine, University of Bari “Aldo Moro”, 70124 Bari, Italy; marialisa.clodoveo@uniba.it

**Keywords:** drugs repositioning, essential oil, antimicrobial resistance, synergism, checkerboard microdilution method, diclofenac

## Abstract

Systemic fungal infections have risen in recent decades and most of them are caused by *Candida* species, which are becoming increasingly resistant to conventional antifungal drugs. Biofilm production has been considered the most common growth form of *Candida* cells and is associated with a high level of antifungal resistance. At present, international research reports on the antifungal activity of non-traditional antimicrobial drugs and their potential use against life-threatening resistant fungal infections. Indeed, drug repurposing has led to the consideration of well-known compounds as a last-line therapy. The goal of this work is to evaluate the potential synergistic antifungal biofilm activity of new combinations between diclofenac sodium salt (DSS), a widely used non-steroidal anti-inflammatory drug (NSAID), with the essential oils (EOs) of *Mentha piperita*, *Pelargonium graveolens*, and *Melaleuca alternifolia*, whose antifungal activity has been well documented over the years. The in vitro antifungal activity of DSS and EOs was determined on different *Candida* strains. Susceptibility testing and the synergism of DSS and EOs versus biofilm cells was performed by using the broth microdilution assay and checkerboard methods. Minimum inhibitory concentrations (sMIC_50_) of DSS alone ranged from 1.25 to 2.05 mg/mL for all the strains considered. These values significantly decreased when the drug was used in combination with the EOs. The fractional inhibitory concentration index (FICI) was lower than 0.5 for almost all the associations, thus indicating a significant synergism, particularly for the DSS–*Pelargonium graveolens* combination towards the *Candida* strains examined. These preliminary results show that the combination of the EOs with DSS improves the antifungal activity on all the tested *Candida* strains, significantly lowering the concentrations of the components used and thus allowing any toxic effects to be overcome.

## 1. Introduction

*Candida* species represent an important source of systemic infections worldwide, widely recognized in modern clinical practice [1]. Over the past ten years, patients with critical illnesses or immunosuppressive conditions have been more susceptible to *Candida* species infections, which also prove more difficult to treat [2]. Considering the growth of immunogenic diseases, the disproportionate use of immunosuppressive drugs, malnutrition, endocrine disorders, the widespread use of indwelling medical devices, and the use of broad-spectrum antibiotics, *Candida* species systemic infections have become one of the leading nosocomial infections at present [3]. Among all the *Candida* species, *Candida albicans* continues to be the most prevalent and problematic one. However, the number of other *Candida* species, non-*Candida albicans Candida* (NCAC) species, identified as being implicated in candidiasis, includes, among others, *Candida glabrata*, *Candida tropicalis*, *Candida parapsilosis*, and *Candida krusei* [4]. The capacity to elude host defenses, the generation of tissue-damaging hydrolytic enzymes, adhesion, and biofilm development (on host tissues and/or medical equipment) are some of the virulence mechanisms that give rise to the pathogenicity of the *Candida* species. The ability of *Candida* species to form drug-resistant biofilms is a significant factor in their contribution to human disease. Biofilm production has been considered as the most common growth form of *Candida* cells and is also associated with the high level of antifungal resistance of the associated microorganisms [3,5]. Current pharmacological therapies for such infections focus on the use of conventional antifungals, such as Amphotericin B, one of the oldest antimycotic agents [6], and drugs belonging to the azoles class [7]. However, the prolonged and widespread usage of these drugs has led to an increase in *Candida* resistance; as a result, there are currently no biofilm-specific antibiotics available on the market that may successfully treat patients’ biofilm infections [8]. Fungal biofilms exhibit a complex and multifactorial resistance to traditional antifungal drugs [9]. Additionally, since several microbial species can cooperate to generate biofilms, they are usually polymicrobial in nature [10]; thus, identifying new strategies to target fungal and polymicrobial biofilm infections is an urgent need [11]. Several drugs from different therapeutic classes are being assessed as antimicrobials in the drug-repositioning approach in an effort to address this problem and find new, affordable solutions [12]. Specifically, it has been reported that a number of non-antibiotic drugs possess antibacterial properties, including statins and non-steroidal anti-inflammatory drugs (NSAIDs), as well as cytostatics and psychotropics [13,14]. NSAIDs are extensively used to treat inflammation and relieve pain. Moreover, recent studies have shown that some of these anti-inflammatory drugs also exhibit antimicrobial activity in addition to their major function [15]. Particularly, since numerous investigations have shown that pathogenic fungi can produce prostaglandins during biofilm adhesion and development, drugs that block the synthesis of prostaglandins, such as NSAIDs and, the most potent, diclofenac, may be essential in influencing the metabolism of fungal prostaglandins. Indeed, several studies reported the effectiveness of NSAIDs against fungal growth both in vitro and in vivo [16]. Furthermore, combination therapies containing NSAIDs and conventional antifungal drugs can alleviate *Candida* spp. biofilm pathologies and other invasive fungal strains. Since plants are a rich source of bioactive secondary metabolites that have been shown to have antifungal effects, extensive research has been recently performed on plant-derived antifungal compounds in an effort to address the problem of antifungal resistance [17]. Among compounds of a natural origin, essential oils (EOs) from aromatic and medicinal plants have received particular attention because of their antibacterial and antifungal activities [18,19]. EOs, like the other phytochemicals, can attenuate the microbial growth and biofilm development through specific mechanisms [20]. One of most significant aspects of EOs’ antimicrobial action is their complex composition characterized by the presence of a ‘universe’ of chemical compounds that can act synergistically. Indeed, the possibility of developing microbial resistance against these complex mixtures of various substances contained in EOs is a remote prospective, since pathogens cannot easily acquire resistance to multiple components [21]. Using an antibiotic agent alongside a natural substance that neutralizes the resistance mechanism is one way to combat antimicrobial resistance and keep the drug effective against resistant microorganisms [22]. In this context, in recent years, we focused our attention on the study of potential synergies between antibiotics or repurposing drugs and EOs, demonstrating the efficacy of these combinations and suggesting the potential of a new therapeutic use [23,24]. Particularly, in our previous work, we demonstrated the synergistic antifungal effect of DSS in combination with *Mentha piperita*, *Melaleuca alternifolia*, and *Pelargonium graveolens* EOs endowed with antimicrobial activity EOs, against the planktonic cells of *Candida* spp. [25]. Starting from the interesting results obtained and given the documented ability of DSS to counteract biofilm growth [26,27,28], the goal of this work is to evaluate if the same combinations are able to synergistically inhibit the biofilm formation of different *Candida* strains.

## 2. Results

### 2.1. EOs’ Chemical Compositions

Gas chromatographic hyphenated with mass spectrometry analyses of EOs were performed following the procedure described in our previous papers [23,25]. *P. graveolens* EO was characterized for 89% of its total composition. The main chemical constituents in a decreasing order were citronellol (26.5%), geraniol (11.7%), γ-eudesmol (7.0%), citronellyl formate (6.9%), linalool (4.7%), and *iso*-menthone (4.6%). *M. piperita* EO was characterized for 96% of its composition. Menthol (35.6%) and *neo*-menthol (9.3%) were the major components. Other compounds present in relevant amount were menthone (24.0%), 1,8-cineole (9.7%), and Δ-3-carene (7.7%). *M. alternifolia* EO was characterized for 83% of the total composition. The major relevant compounds were terpinen-4-ol (33.4%), γ-terpinene (17.2%), aromandrene (4.4%), ledene (3.9%), and α-terpinolene (3.8%). A detailed description of the EOs’ phytochemical compositions was reported in our previous work [25].

### 2.2. Antifungal Activity

In this research, different EOs were used in combination with DSS to inhibit yeast biofilm growth. In particular, the effects of these combinations were evaluated on seven fungal ATCC strains producing biofilm. Table 1 illustrates the effects of these associations (EOs–DSS) and reports the reductions in the growth of percentage values together with the quantities of each component in the association at the most effective inhibition growth. Furthermore, all the minimal inhibitory concentration values (sMIC_50_) for each EO and DSS were reported for comparison. The FIC index (FICI), a parameter that studies the synergism of two compounds, was reported too. The best results were obtained with the DSS–*Pelargonium graveolens* EO association with an inhibition percentage of the biofilm mass in the range of 75.41–54.76%. For the DSS-*Mentha piperita* EO association, the inhibition percentage was in the range of 75.16–57.14%, while for the DSS-*Melaleuca alternifolia* EO association, the range was 55.23–43.00%. It is worth noting that the quantities of DSS able to inhibit biofilm growth were remarkably decreased when combined with EOs (Table 1). Indeed, the sMIC_50_ of the DSS ranged between 1.25–2.05 mg/mL, whereas the concentration of DSS in association, at the most effective combination, ranged between 0.10–082 mg/mL with *M. piperita*, and 0.1–0.4 mg/mL in association with *P. graveolens*. On the other hand, the concentration of DSS in the association with *M. alternifolia* was in the 1.25–2.05 mg/mL range, similar to its sMIC_50_. In particular, the reduction in concentrations for DSS associated with *P. graveolens* EO showed a remarkable decrease ranging between 5–20 times for all the strains tested, being the best result obtained for *C. krusei* ATCC 14243. Similarly, the DSS–*M. piperita* EO association showed interesting results with a reduction range of 2.5–20 times. For this association the greater reduction in the DSS concentration was obtained against *C. krusei* ATCC 6528. A lower trend was observed for the DSS-*M. alternifolia* association for which the concentrations reduction ranged between 2.5–5 times. Interestingly, also concentrations of the EOs in the mixture at the most effective combination were significantly lower than the corresponding sMIC_50_ values. Particularly noteworthy is the result obtained with DSS-*M. piperita* EO for *C. kefir* ATCC 204093, being the active concentration of the EO in the association 32-fold lower than the corresponding sMIC_50_ value. As regards the FICI vales, they ranged between 0.30–0.45 for the DSS-*M. piperita* EO association, these values being lower than the limit value of 0.5 and confirming the existence of a strong synergism between the NSAD and EO. The best results were registered for *C. krusei* ATCC 6528 and *C. tropicalis* ATCC 750 with FICI values of 0.30 for both. Similarly, for DSS-*P. graveolens* EO, the range was between 0.23–0.45, the higher synergism being observed for *C. tropicalis* ATCC 750 with an FICI value of 0.23. Conversely, for DSS-*Melaleuca alternifolia* EO, only two strains showed a significant FIC index equal to 0.45: *C. krusei* ATCC 14243 and *C. kefir* ATCC 204093.

## 3. Discussion

**Figure 1:**
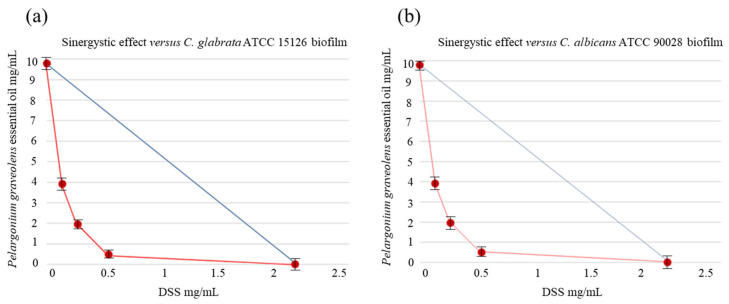
Isobole curves revealing the synergistic effect of DSS with *Pelargonium graveolens* EO on inhibiting (**a**) *Candida glabrata* ATCC 15126 and (**b**) *Candida albicans* ATCC 90028 biofilms. The non-interaction of the two components results in a straight line, whereas the occurrence of an interaction is shown by a concave isobole. Bars represent medium ± standard deviation values obtained from three individual experiments performed in triplicate.

**Figure 2:**
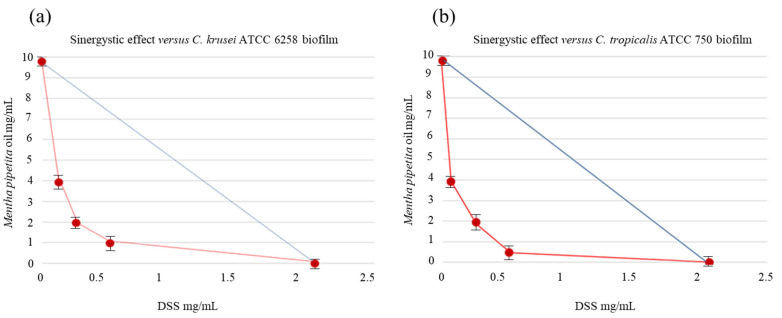
Isobole curves revealing the synergistic effect of DSS with *Mentha piperita* EO on inhibiting (**a**) *Candida krusei* ATCC 6258 and (**b**) *Candida tropicalis* ATCC 750 biofilms. The non-interaction of the two components results in a straight line, whereas the occurrence of an interaction is shown by a concave isobole. Bars represent medium ± standard deviation values obtained from three individual experiments performed in triplicate.

Due to their ability to adapt to environmental stressors, host immunological responses, and conventional antimicrobial treatments, biofilms are the preferred growth method for most microorganisms, including several fungal pathogens belonging to the *Candida* species [29]. Indeed, some classical antifungals agents generally display a 1000-fold increase in in vitro sMIC towards biofilm-forming fungal cells, in comparison to planktonic cells. Because of the multidrug-resistance mechanisms found in fungal biofilms, the currently available antifungal drugs are unable to effectively eradicate biofilm-associated infections and, consequently, new strategies to overcome this problem are desirable. In this context, the drug repositioning approach comprises a promising trend in the field of antimicrobial drug discovery by identifying new therapeutic opportunities among existing drugs. NSAIDs are an important class of anti-inflammatory drugs widely used for the treatment of musculoskeletal disorders, mild-to-moderate pain, and fever; however, these drugs have been shown to possess antifungal activities against different strains of *Candida*, in addition to their main activity. The inhibition of fungal PG synthesis can be one of the possible mechanisms of NSAIDs responsible for preventing fungal growth and biofilm development and adhesion in *Candida* species [16]. The combination of repositioned drugs with EOs endowed with well-documented antimicrobial properties can be an interesting approach for the rapid identification of new therapies to treat acute infections. In a recent study, we demonstrated the synergistic antifungal effect of DSS, in combination with EOs endowed with antimicrobial activity, towards different strains of *Candida* spp. [25]. In this study, we went beyond the study of planktonic cells and reported the inhibitory effect of the same associations against the biofilm-forming *Candida* species reported in Table 1. As underlined in our in vitro assays, the fungal strains under study displayed their sensitivity to the compounds tested, both individually and in combination, showing the DSS-EOs association’s significant reduction in the biofilm mass of the *Candida* species considered. These promising findings allowed us to assess the synergistic effect between DSS and EOs against *Candida* spp. biofilms, which was verified for almost all the associations tested, as shown by the FIC indexes of the DSS-EOs associations reported in Table 1. Indeed, the FICI values were, in most cases, lower than the limit value of 0.5, being the best results obtained for the DSS-*Pelargonium graveolens* EO association. Particularly interesting was the reduction in the concentration required to inhibit the biofilm growth of DSS alone when compared to that of DSS in combination. This result was particularly noteworthy in the case of the association with *Pelargonium graveolens* EO against *Candida tropicalis* ATCC 750. Indeed, the use of DSS alone required 2.05 mg/mL, whereas, when it was combined with *Pelargonium graveolens* EO, only 0.21 mg/mL was sufficient to provide a biofilm growth reduction amounting to 75.41 ± 0.31%. This clear-cut synergistic effect was confirmed by a corresponding FICI value of 0.23, significantly lower than the limit value of 0.5. Another indicative example was the inhibitory effect against the *Candida krusei* ATCC 6528 biofilm of the DSS-*Mentha piperita* EO association. In fact, the use of DSS alone required 2.05 mg/mL, whereas, when it was combined with the EO, only 0.10 mg/mL was sufficient to provide a biofilm growth reduction amounting to 75.16 ± 0.24%. The observed activities could be linked to the phytochemical profiles of the EOs analyzed by GC-MS. The active components of EOs, including monoterpene alcohols (e.g., citronellol, geraniol, menthol, and terpinene-4-ol), epoxides (e.g., 1,8-cineole), ketones (e.g., *iso*-menthone), and hydrocarbons (e.g., γ-terpinene and Δ-3-carene) collaborated with the DSS to exert their effects. The bioactivities could also be attributed to the minor constituents present in the EO compositions belonging to the monoterpene and sesquiterpene chemical classes, including both hydrocarbons and oxygenated compounds, which synergistically supported the main chemical constituents in the multifactorial antifungal mechanism [30,31]. Indeed, the mechanism of action may involve multiple factors and result from the intricate interplay of the constituent parts. According to information found in numerous academic publications, the synergy of EOs can be attributed to their capacity to compromise the permeability barrier of the microbial plasma membrane [30]. This disruption might make it easier for the DSS to enter the microbial cell, interact with the COX mechanisms, and then exert its antifungal effects. It was an important claim that a reduction in the mature biofilm was obtained with lower concentrations of the two components. These findings are represented by the isobole curves (Figure 1 and Figure 2) that illustrate four of the most effective synergistic results.

## 4. Material and Methods

### 4.1. Materials

#### 4.1.1. Essential Oils

EOs from *Mentha piperita* L. leaves (LOT F011023, 10/2023), *Pelargonium graveolens* L’Hér. leaves (LOT F810074, 07/2022), and *Melaleuca alternifolia* (Maiden & Betche) Cheel leaves (F911010, 04/2024) were obtained by hydrodistillation and were kindly provided by Puressentiel Italia (Milan, Italy). The EO samples already subjected to gas chromatographic analysis (GC-MS) in our previous investigation [25] were stored and protected from light and humidity in brown, glass bottles at the temperature of 0–4 °C until the testing analysis or microbiological assays were performed.

#### 4.1.2. Chemicals

The DSS was purchased from Farmalabor (Canosa di Puglia—Bari, Italy). The culture media used were Sabouraud 2% dextrose broth (Oxoid, Rodano, Italy) and Yeast Malt Broth (Oxoid, Rodano, Italy).

#### 4.1.3. Fungal Strains

The antifungal activity was tested against many fungal strains and included different strains belonging to the American Type Culture Collection (ATCC, Rockville, MD, USA) that were *C. albicans* (ATCC 10231), C. albicans (ATCC 90028), *C. glabrata* (ATCC 15126), *C. tropicalis* (ATCC 750), C. kefyr (ATCC 204093), and *C. krusei* (ATCC 6258).

### 4.2. Methods

#### 4.2.1. Fungal Strains and Antifungal Testing

A total of yeast strains were used in this study: *Candida albicans* ATCC 10231, *Candida albicans* ATCC 90028, *Candida glabrata* ATCC 15126, *Candida kefyr* ATCC 204093, *Candida krusei* ATCC 14243, *Candida krusei* ATCC 6258, and *Candida tropicalis* ATCC 75. Strains were maintained at −80 °C in yeast peptone dextrose broth with 10–25% glycerol (Oxoid, Rodano, Italy) solution. All strains were stored at −20 °C in glycerol stocks and were subcultured on antimicrobial agent-free Sabouraud Dextrose Agar plates (BioMerieux, Marcy L’Etoile, France) to ensure viability and purity before the start of the study.

#### 4.2.2. Medium and Culture Conditions

Each frozen stock culture was inoculated in Sabouraud Dextrose Broth (Difco, Milan, Italy) and incubated at 37 °C for 24 h in an orbital shaker at 60 rpm. Cells were picked up and added to a tube containing RPMI 1640 broth medium with L-glutamine and without bicarbonate buffered to pH 7 with MOPS, 3-(N-morpholino) propane sulfonic acid (165 M, Sigma, Milan, Italy). A standardized suspension of 1 × 10^6^ CFU/mL was obtained and immediately used.

#### 4.2.3. Preparation of the Test Solution

The EOs were solubilized in ethanol in 1:5 proportions and then diluted in RPMI 1640 broth medium added with tween 80. The DSS was solubilized in DMSO and, subsequently, in a culture medium.

#### 4.2.4. Biofilm Biomass Measurement and Reduction

To evaluate the synergistic anti-biofilm action of EOs in association with the DSS, we performed the in vitro colorimetric XTT (2,3-bis(2-methoxy-4-nitro-5-sulphophenyl)-5-[(phenylamino)carbonyl]-2*H*-tetrazolium hydroxide) assay. Briefly, 200 µL of a yeast culture (10^6^ cfu/mL) was added to each well of a 96-well flat microtiter plate and incubated for 24 h at 37 °C by shaking on a rocker table to allow cell attachment and biofilm formation. Then, 200 µL of antibiotic was added as a positive control, while the negative control contained only RPMI 1640 instead of a DSS-EOs association. Following incubation, the contents of each well were removed and the wells were rinsed with 100 µL of sterile PBS to remove loosely attached cells and non-adherent and nonviable cells. Following incubation, 200 µL of each EO-DSS combination was added to the wells. Four double serial dilutions of the 40% Ethanol EO, with Tween 80 0.1%, were prepared following the same method used to evaluate the MIC described in our previous works. Dilutions of the EOs were prepared together with a series of double dilutions of the drugs and EOs. This method was used to mix all the diclofenac dilutions with the appropriate concentrations of EOs so that a series of concentrations combinations of the DSS-EOs being considered were obtained [32]. The concentrations prepared accounted for 40%, 20%, 10%, and 5% of the MIC values for the EO, and 25%, 12.5%, 6.25%, and 3.12% of the MIC values for diclofenac [33,34]. After the incubation period with the drug, the media was removed from each well and supports were washed with 1 mL of PBS. The biofilms were then plunged in 2 mL of PBS with the addition of 180 μL of XTT solution (1 mg/mL) and Menadione (0.4 mM) at a ratio of 6:1. This solution was prepared by solubilizing XTT in sterile water (obtained by filtration) and Menadione in dimethyl sulfoxide. The plate with supports was incubated for 2 h at 37 °C. After 2 h, the plate was read with a microplate spectrophotometer (Perkin-Elmer Wallac Victor3 Microplate reader, Perkin-Elmer, Milan, Italy) at a 490 nm wavelength, where there was a maximum Formazan absorbance peak. This is a little-used method in international testing, although it is the most useful, thanks to the fact that it needs volumes well below the 24-wells method, going from milliliters to microliters and producing a considerable cost reduction. The sessile minimal inhibitory concentration (sMIC) was the lowest anti-biofilm concentration for both compounds causing a visible reduction in the growth biofilm compared with the growth in the control well, without the presence of diclofenac or EO [35]. The percentage of reduction in the biofilm obtained with the combinations studied was determined as follows: percentage of biofilm reduction = (OD control well − OD experimental)/(OD control well) × 100 [36]. Antimicrobial susceptibility tests of the biofilms were performed in triplicate on different days. In our experimental protocols, the combinations of the substances were analyzed by calculating the FIC index (FICI). Generally, the FICI value was interpreted as (i) a synergistic effect when it was ≤0.5; (ii) an additive or indifferent effect when it was >0.5 and <1; and (iii) an antagonistic effect when it was >1 [24]. The findings are presented as the means ± SDs of at least three distinct measurements performed in triplicate.

## 5. Conclusions

The synergistic associations of drugs represent a valid approach to antimicrobial therapies, since they have provided positive results in recent years. Our previous studies on EOs, based on the synergy with DSS, demonstrated the effectiveness of these associations on the planktonic cells of *Candida* spp. Since several studies report that microbial biofilms are the basis of many persistent diseases and one of the main mechanisms used by microorganisms to give rise to resistance, the goal of this study is the in vitro evaluation of the synergistic effects of DSS with different EOs (*Melaleuca alternifolia*, *Mentha piperita*, and *Pelargonium graveolens*) against the *Candida* spp. biofilm at different concentrations, using the XTT microtiter method and FIC index. It has been shown that the well-known prostaglandin synthesis inhibitor diclofenac, in combination with EOs, greatly reduces biofilm production by several species of *Candida* spp. ATCC. An important achievement is the significant reduction in EOs and DSS quantities when their associations were tested against biofilms. The rational analysis of bioactivity outcomes correlated with the phytochemical composition allowed us to highlight the most promising EO profiles for the synergistic association with drugs, such as DSS. In particular, concerning the DSS-*P. graveolens* association, the concentration decrease ranged from 5 to 20 times. Furthermore, the association between DSS and EOs considered in our research markedly reduced the viability of biofilm cells up to values of about 75% for some of the *Candida* strains considered. However, the main limitation of this study lay in being a preliminary in vitro analysis. Further investigations will provide further knowledge of the mechanism underlying the synergism and a more complete understanding of the antimicrobial potential of these associations. In addition, further in-depth studies will be needed to create ad hoc formulations of the association so that clinical use can be considered. In conclusion, our results may represent an interesting starting point for an alternative route to new synergistic antifungal therapies against biofilm infections, overcoming the high cost of new drugs, the potential risk of antagonistic interactions, and limit the use of classical antibiotics to which microorganisms are presently increasingly resistant.

## Figures and Tables

**Table 1:** Inhibitory effects (% Red) of different essential oils (EOs), diclofenac sodium salt (DSS), and their combinations on mature fungal biofilms.

		EO mg/mL		DSS mg/mL		Synergism				Anidulafungin µg/mL	
Strains	Essential Oil	sMIC_50_^a^	%Red ± SD ^b^	sMIC_50_^c^	%Red ± SD ^d^	EO mg/mL ^e^	DSS mg/mL ^f^	DSS + EO %Red ± SD ^g^	FICI ^h^	sMIC_5_^i,c^	%Red ± SD ^l^
*C. albicans* ATCC 10231	*M. alternifolia*	9.4	51.00 ± 0.82	1.25	40.00 ± 0.82	1.2	0.5	43.00 ± 0.82	0.53	2.00	33.98 ± 0.41
	*M. piperita*	4.9	68.00 ± 2.04			1.23	0.25	60.77 ± 0.21	0.45		
	*P. graveolens*	9.6	45.50 ± 0.85			2.40	0.13	58.87 ± 0.64	0.35		
*C. albicans* ATCC 90028	*M. alternifolia*	9.4	53.00 ± 0.47	1.25	63.00 ± 1.25	2.4	0.5	48.47 ± 0.41	0.65	2.00	16.09 ± 0.21
	*M. piperita*	9.8	45.50 ± 0.85			2.45	0.25	66.27 ± 0.52	0.45		
	*P. graveolens*	9.6	64.80 ± 0.08			1.20	0.3	74.27 ± 0.21	0.33		
*C. glabrata* ATCC 15126	*M. alternifolia*	9.4	44.30 ± 0.92	2.05	52.05 ± 0.76	2.35	0.82	48.80 ± 0.22	0.65	2.00	27.41 ± 0.64
	*M. piperita*	9.8	49.30 ± 0.17			2.45	0.41	74.10 ± 0.12	0.45		
	*P. graveolens*	9.6	62.44 ± 1.29			1.20	0.21	56.02 ± 0.73	0.23		
*C. kefyr* ATCC 204093	*M. alternifolia*	4.7	45.50 ± 0.39	2.05	53.24 ± 1.13	1.18	0.41	45.60 ± 0.43	0.45	2.00	51.24 ± 0.41
	*M. piperita*	9.8	68.00 ± 0.71			0.31	0.82	57.14 ± 0.69	0.43		
	*P. graveolens*	9.6	46.00 ± 0.85			1.20	0.41	58.50 ± 0.41	0.33		
*C. krusei* ATCC 14243	*M. alternifolia*	9.4	44.00 ± 0.47	2.05	42.00 ± 0.82	2.35	0.41	50.00 ± 0.82	0.45	2.00	47.51 ± 0.88
	*M. piperita*	4.9	55.60 ± 2.05			0.61	0.41	65.67 ± 0.34	0.33		
	*P. graveolens*	4.8	68.00 ± 0.95			1.20	0.10	54.76 ± 0.88	0.30		
*C. krusei* ATCC 6528	*M. alternifolia*	9.4	46.00 ± 4.75	2.05	41.33 ± 0.47	2.35	0.82	55.23 ± 1.60	0.65	2.00	28.64 ± 0.30
	*M. piperita*	4.9	44.00 ± 2.50			1.23	0.10	75.16 ± 0.24	0.30		
	*P. graveolens*	9.6	53.00 ± 0.94			2.40	0.41	55.65 ± 1.71	0.45		
*C. tropicalis* ATCC 750	*M. alternifolia*	4.7	4.00 ± 0.54	2.05	46.00 ± 0.82	0.59	0.82	55.20 ± 0.51	0.53	2.00	32.44 ± 0.67
	*M. piperita*	9.8	63.76 ± 0.87			2.45	0.10	57.70 ± 0.36	0.30		
	*P. graveolens*	4.8	76.10 ± 0.29			0.60	0.21	75.41 ± 0.31	0.23		

sMIC_50_^a^: EO sessile minimal inhibitory concentration that inhibited at least 50% of the metabolic activity; %Red ± SD ^b^: EO biofilm mass inhibition rate ± standard deviation; sMIC_50_^c^: DSS sessile minimal inhibitory concentration that inhibited at least 50% of the metabolic activity; %Red ± SD ^d^: DSS biofilm inhibition rate ± standard deviation; EO mg/mL ^e^: concentration of the essential oil in the mixture at the most effective combination; DSS mg/mL ^f^: concentration of DSS in the mixture at the most effective combination; DSS + EO %Red ± SD ^g^: biofilm combination mixture inhibition rate; FICI ^h^: fractional inhibitory concentration index; sMIC_50_^i^: anidulafungin (reference drug) sessile minimal inhibitory concentration that inhibited at least 50% of the metabolic activity; %Red ± SD ^l^: anidulafungin biofilm mass inhibition rate ± standard deviation; DSS: Diclofenac sodium salt; EO: essential oil.

## Data Availability

The data is contained within the manuscript.

## Abbreviations

DSS, Diclofenac Sodium Salt; EOs, Essential Oils; FICI, Fractional Inhibitory Concentration Index; GC, Gas Chromatography; MS, Mass Spectrometer; MIC, Minimal Inhibitory Concentration; sMIC, Sessile Minimal Inhibitory Concentration; SD, Standard Deviation.
